# Reflex and reflective testing: progress, but much still to be done

**DOI:** 10.1177/0004563221993153

**Published:** 2021-02-10

**Authors:** Michael J Murphy

**Affiliations:** Department of Biochemical Medicine, Ninewells Hospital & Medical School, Dundee, UK

Clinical biochemists add value collectively by participating with other health professionals in the delivery of a high-quality service. They may also add value individually in various ways, including clinical liaison, adding comments and adding tests. Of these activities, the last is the most amenable to quantification; the metrics for adding value by adding tests are conceptually simple and readily applied. Reflex testing (the automatic addition of tests by analysers based on algorithms established by laboratory professionals) necessarily does most of the ‘heavy lifting’, reflecting the enormous throughput of heavily automated contemporary NHS laboratories. A much smaller number of tests is added reflectively by clinical biochemists. Paradoxically, reflective testing has received more attention, with early studies^1^,^2^ in particular seeking to prove the principle that it identifies patients who would otherwise be missed. However, subsequent studies^3^,^4^ have tended to report on both reflex and reflective testing. This is just as well, since they are indissolubly linked – and the impact of the choice of reflex threshold on the metrics of both has received inadequate attention.

The *Annals* has recently published a couple of papers which add significantly to what we know about the practice and value of adding tests. The first is a survey of reflex and reflective testing practice in the United Kingdom (UK).^5^ This provides a detailed snapshot of current practice across a range of commonly encountered diagnostic scenarios, and perhaps its most useful function is to set out the landscape for how tests are added. Significant variation in practice was observed across a range of activities and scenarios. Examples include: whether or not a test is added; if so, whether it is added reflectively or reflexly; whether interpretive comments are included; whether the requestor is contacted before addition of tests. The results of the survey thus provide a useful benchmarking function for laboratory professionals. They also provide a valuable first step in laying the foundation for identifying best practice in this area; as the authors highlight, such advice exists for interpretation^6^ even though as pointed out above this is less readily measured.

A few observations are warranted. First, the thresholds used to trigger reflex addition of tests vary widely, e.g. the hypocalcaemic threshold to trigger magnesium measurement varied from 1.50 mmol/L up to 2.20 mmol/L. Even allowing for differences in the nature, size and staffing of hospital laboratories, and populations served, the extent of the observed variation invites scrutiny. Second, in the table which documents quantitative aspects of the survey, reflective thresholds are listed alongside the reflex thresholds for each scenario, as if equivalent. However, reflective ‘thresholds’ must be interpreted cautiously, for two reasons: (a) the *raison d’être* of reflective testing is that it permits more complex information to be taken into consideration than can readily be incorporated into reflex algorithms^7^ – thus any ‘threshold’ is of its nature ‘softer’ – if ‘hard’, it is effectively a reflex threshold; (b) the reflex thresholds/boundaries used in each diagnostic scenario affect the clinical utility of reflective testing.^4^ Third, the addition of tests is widely accompanied by interpretive comments, although it is not clear from the results of the survey how these were broken down by reflex/reflective testing, or where these were automated. In general, one might expect automated comments to accompany reflexly added tests, and non-automated comments to be used to explain the more complex rationale of reflective testing. In both cases, the addition of tests and comments is closely entwined. Finally, notwithstanding ethical issues in the addition of some tests, e.g. diagnosis of malignancy or pregnancy, it is clear that different approaches are used in the binary decision to contact the requestor before adding the test, or not.

This issue sees the print publication of a second important paper in this area: a randomised controlled trial (RCT) of reflective testing in primary care patients.^8^ In this study, patients were randomly allocated to an intervention group, where requesting clinicians received reflectively added test results and interpretive comments as appropriate, in addition to the originally requested tests, and a control group where they did not. The medical records of patients in each group were followed up six months after the reports/comments were issued. Primary outcome measures were the adequacy or otherwise of intended and actual actions, as judged by a multidisciplinary panel. Reflective testing was judged to be useful in 84% of cases, and favourably shifted the distribution of adequate/neutral/inadequate actions.

As the first RCT of reflective testing, this study is a landmark step towards providing an evidence base for adding tests. The outcomes were appropriately nuanced, accommodating a spectrum of adequacy and the elapsed interval post test/comment enough to remove any possibility of confounding due to premature evaluation. The adjudicating panel was broadly based, including physicians from both primary and secondary care, as well as a clinical chemist. The delta checks and thresholds are supplied in supplemental material. In short, this was a well-designed and executed study. A significant omission was the failure to document the efficiency and effectiveness of the reflective testing in each group. In a study where the other outcomes were ‘softer’ or at least more subjective, this would have provided helpful quantitative detail.

This was, strictly, not an RCT of reflective testing; rather, it was an RCT of reflective testing and associated interpretive comments. As highlighted above and elsewhere,^9^ it is much harder to quantitate the value of an interpretive comment than an added test; evaluating the combined effect of both activities ‘muddies the methodological waters’. However, since these activities are so closely related, some may judge that it is appropriate to evaluate them together. Second, the finding that reflective testing was judged to be useful, and added value, was not surprising. It would have been astonishing, and deeply troubling, if this were not the case. Third, as the authors acknowledge, there was a significant – and unexplained – difference between intervention and control groups in terms of consenting to participate, allowing for the possibility of selection bias. Finally, the reports selected for potential reflective testing were prefiltered by computer algorithms/delta checks, allowing for a different kind of selection bias.

**Figure 1.**
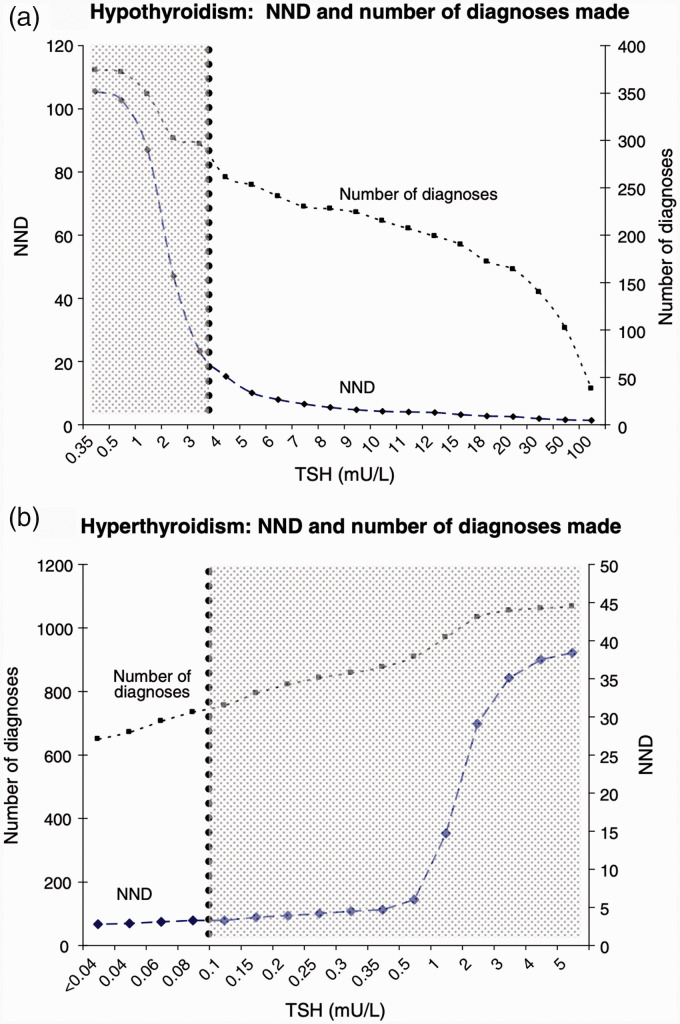
Number of diagnoses (cumulative) and numbers needed to diagnose (NND) for (a) hypothyroidism and (b) hyperthyroidism. Vertical dotted lines indicate reflex thresholds. Reflective testing is confined to shaded areas. Reproduced with permission from Murphy.^9^ TSH: thyroid-stimulating hormone.

What needs to be done next? The most pressing issue is to establish the variation in efficiency and effectiveness across the range of reflex thresholds used in each diagnostic scenario. The variation in these metrics by threshold has been established for TSH triggering free thyroxine measurement,^4^ but a comprehensive evaluation is required covering other commonly encountered diagnostic scenarios. (Essentially, the data shown in Figure 1 for free thyroxine triggered by TSH need to be replicated for other triggering/triggered tests). The work involved is not trivial, but this is the first step to establishing optimal thresholds to apply. It cannot be assumed that these metrics will vary in the same way for different scenarios. The process of generating these data will reinforce awareness of the effect of reflex thresholds on the metrics of both reflex and reflective testing. It will also provide the basis for establishing best practice in the addition of tests. Establishing ‘best practice’ in the absence of data carries the risk of ‘fossilising’ activities which are not evidence-based (laboratory professionals would require compelling reasons not to adhere to guidance issued by national professional bodies). Reflective testing in particular is a discretionary activity and it is important that we retain individual professional autonomy.
